# Development of a Social Network for People Without a Diagnosis (RarePairs): Evaluation Study

**DOI:** 10.2196/21849

**Published:** 2020-09-29

**Authors:** Lara Kühnle, Urs Mücke, Werner M Lechner, Frank Klawonn, Lorenz Grigull

**Affiliations:** 1 Hannover Medical School Hannover Germany; 2 KIMedi GmbH Donauwörth Germany; 3 Helmholtz Centre for Infection Research Braunschweig Germany; 4 Ostfalia University Wolfenbüttel Germany; 5 University Hospital Bonn (UKB) Bonn Germany

**Keywords:** rare disease, diagnostic support tool, prototype, social network, machine learning, artificial intelligence

## Abstract

**Background:**

Diagnostic delay in rare disease (RD) is common, occasionally lasting up to more than 20 years. In attempting to reduce it, diagnostic support tools have been studied extensively. However, social platforms have not yet been used for systematic diagnostic support. This paper illustrates the development and prototypic application of a social network using scientifically developed questions to match individuals without a diagnosis.

**Objective:**

The study aimed to outline, create, and evaluate a prototype tool (a social network platform named RarePairs), helping patients with undiagnosed RDs to find individuals with similar symptoms. The prototype includes a matching algorithm, bringing together individuals with similar disease burden in the lead-up to diagnosis.

**Methods:**

We divided our project into 4 phases. In phase 1, we used known data and findings in the literature to understand and specify the context of use. In phase 2, we specified the user requirements. In phase 3, we designed a prototype based on the results of phases 1 and 2, as well as incorporating a state-of-the-art questionnaire with 53 items for recognizing an RD. Lastly, we evaluated this prototype with a data set of 973 questionnaires from individuals suffering from different RDs using 24 distance calculating methods.

**Results:**

Based on a step-by-step construction process, the digital patient platform prototype, RarePairs, was developed. In order to match individuals with similar experiences, it uses answer patterns generated by a specifically designed questionnaire (Q53). A total of 973 questionnaires answered by patients with RDs were used to construct and test an artificial intelligence (AI) algorithm like the k-nearest neighbor search. With this, we found matches for every single one of the 973 records. The cross-validation of those matches showed that the algorithm outperforms random matching significantly. Statistically, for every data set the algorithm found at least one other record (match) with the same diagnosis.

**Conclusions:**

Diagnostic delay is torturous for patients without a diagnosis. Shortening the delay is important for both doctors and patients. Diagnostic support using AI can be promoted differently. The prototype of the social media platform RarePairs might be a low-threshold patient platform, and proved suitable to match and connect different individuals with comparable symptoms. This exchange promoted through RarePairs might be used to speed up the diagnostic process. Further studies include its evaluation in a prospective setting and implementation of RarePairs as a mobile phone app.

## Introduction

A patient without a diagnosis desperately struggles for help. This holds especially true for those with an undiagnosed rare disease (RD). Although an RD is one that, by definition, only 5 out of 10,000 people suffer from, in total there are approximately 13.5 million people with an RD in the European Union (EU) [1] and approximately 400 million worldwide [2]. Affected patients search for the diagnosis for an average of 8 years. During this time, misdiagnosis and wrong treatments are common, and social isolation and financial damage occur frequently [3-9]. By contrast, patients with a diagnosed RD are highly active in supporting each other, and may serve as experts for their diseases in patient groups. This is an important resource for information and guiding besides the information on RD in the internet.

The internet has grown to be an easily accessible hub for research, even for health care information. Today, almost everybody is *Googling* symptoms before, while, and after a health care visit [10]. The power of internet-based diagnosis was recently underscored by Siempos et al [11], highlighting that 22.1% of correct diagnoses from laymen were due to web searches. Furthermore, doctors themselves similarly consult the internet searching for the correct diagnosis [12]. Here, in 58% of the cases, search engines such as Google helped identify the diagnosis [12].

Besides searching the internet, almost all young Americans aged between 18 and 29 use social media [13]. Communicating via those networks is a daily activity for them, and using social media platforms has become an established way of making personal connections [14]. Online social networks are not tied to a specific time or place, making them even more efficient for communication. There are online social networks that help preserve contacts over distances, as well as networks facilitating meeting people with common interests or issues. Those networks often use matching algorithms containing artificial intelligence (AI) to match for optimum results. Moreover, such matching algorithms are used in marketing to find products and services that fit the needs of a person.

In *RarePairs* we also use a matching algorithm to meet the needs of potential users. This prototype of a new social platform is designed to bring people with and without a diagnosis together, making interaction and supporting to find the right diagnosis possible. Thus, it tries to help find the right people to discuss possible diagnoses, coping strategies, and treatments for the user’s symptoms. We decided to focus on the group of RDs as they are still overlooked in cases of diagnosis, care, and treatment. The idea behind RarePairs is to combine already existing resources (social networks, the internet, smart mathematical algorithms, an existing questionnaire/data set), use cases (finding diagnoses, health information, contact to other people with the same condition), and challenges (diagnostic odyssey, a very small global proportion of people with the same condition), and fit them into one tool. The aim of this study was to outline, create, and evaluate a prototype of RarePairs.

## Methods

To ensure the quality of the design process for an online social network, we used an ISO norm. This ISO norm is designed to develop a user-centered design software product.

To build our prototype we used ISO 9241-210:2010 and followed the suggested 4 steps: (1) Understand and specify the context of use; (2) Specify the user requirements; (3) Produce design solutions to meet these requirements; and (4) Evaluate the designs against requirements.

To complete steps 1 and 2, we collected known facts based on different materials, such as the German website for information on RDs [15]. Additionally, we used expert knowledge about people with RD which was collected from previous research and discussions with patient groups. Details on the completed steps can be found in Multimedia Appendix 1.

In the second step, this information was discussed with an interdisciplinary team of doctors, computer scientists, and mathematicians to define the context of use and user requirements. To complete step 3, we used commonly known hardware and software (all-day-use laptops, *Adobe Photoshop*, Text editors, *MAMP*, and *GitHub*) for the web design of our prototype. We also used common coding formats, such as HTML, CSS, PHP, MySQL, and R, for designing the prototype. No templates or content management systems were used.

For the most important part of the prototype, the matching algorithm, we resorted to a questionnaire named Q53 which was built during previous research in the working group [16]. Briefly, this questionnaire was built using patients’ experience. Individuals with different RDs were interviewed to gain insight into their prediagnostic experiences. These experiences (in daily life) were qualitatively analyzed. In a 7-step process following strict rules we finally ended up with a set of 53 questions. Afterward, larger cohorts of individuals with different RDs (and established diagnoses) were contacted and invited to answer the questions. This approach was based on the idea that most people with different RDs not only experience similar basic symptoms (eg, fatigue, blaming) during their prediagnostic *odyssey*, but also have comparable strategies on finding a diagnosis (eg, consulting various doctors) or coping with daily life (eg, avoidance strategies, intuitive usage of assistive technologies). Based on these experiences from the period (sometimes years) prior to the diagnosis, which were collected through interviews from individuals with a proven RD, 53 questions were identified as being crucial and prototypic for individuals with different RDs. The 53 questions break down into 7 different categories (eg, symptoms, social environment, or looking for the cause), and can be answered with: (1) *No*, (2) *Slightly no*, (3) *Slightly yes*, (4) *Yes*, and (5) *Do not know*. Thus, the questionnaire Q53 not only asks for specific symptoms, but also reflects the challenges and obstacles of individuals with different RDs in daily life, typical circumstances, and certain actions, and the Q53 can be answered without expert knowledge within 15 minutes. Some examples out of the set of 53 questions are (1) Do you withhold information about your complaints from your environment (eg, family, friends, colleagues)? (2) Do you use supportive devices to positively help your daily routine; (3) Did you suspect—if so since longer—that something is “wrong” with you? (4) Do you use tricks and dodges to master restrictions during your daily life? (5) Would you say that the ambiguity about the cause of your complaints/irritating phenomena was the worst? (see Multimedia Appendix 2 [German] or Multimedia Appendix 3 [English] for the whole Q53 questionnaire).

In the previous study [16] which described the development of the questionnaire, the set of questions was then used by a matching algorithm combining the results of support vector machine, random forest, logistic regression, and linear discriminant analysis, to effectively classify a data set of approximately 1000 questionnaires of individuals with different RDs and other disease conditions into 4 different diagnostic categories [16]. Briefly, these diagnostic categories differentiated between RDs, chronic diseases (CDs), psychosomatic disorders, and other disease conditions. Hence, the questionnaire Q53 was originally *not* designed for diagnosing a specific disease, but it proved effective to cluster diseases into diagnostic categories (which might also prove helpful for guiding individuals during a *diagnostic odyssey*). As the aim of RarePairs is bringing together undiagnosed/diagnosed people so as to share their symptoms and coping strategies, such a questionnaire might suit well for this purpose.

In the second step, an existing data set (from previous study [16]) of answered Q53 questionnaires (n=973) from individuals with non-RDs and different RDs, such as neuromuscular diseases (eg, Pompe disease, amyotrophic lateral sclerosis), autoimmune diseases (eg, sarcoidosis, systemic lupus erythematosus), or rare metabolic diseases (eg, glycogen storage diseases), was used for prototypic evaluation of RarePairs.

Of the 973 people, 759 were previously and certainly diagnosed with an RD, 27 were healthy, 34 had an unknown diagnosis, 27 were diagnosed with psychosomatic disorders, and 126 had a CD. They were between 0 and 87 years of age with a mean age of 39.4 years (702 were female and 271 were male). Recruitment was limited to Germany.

**Figure 1:**
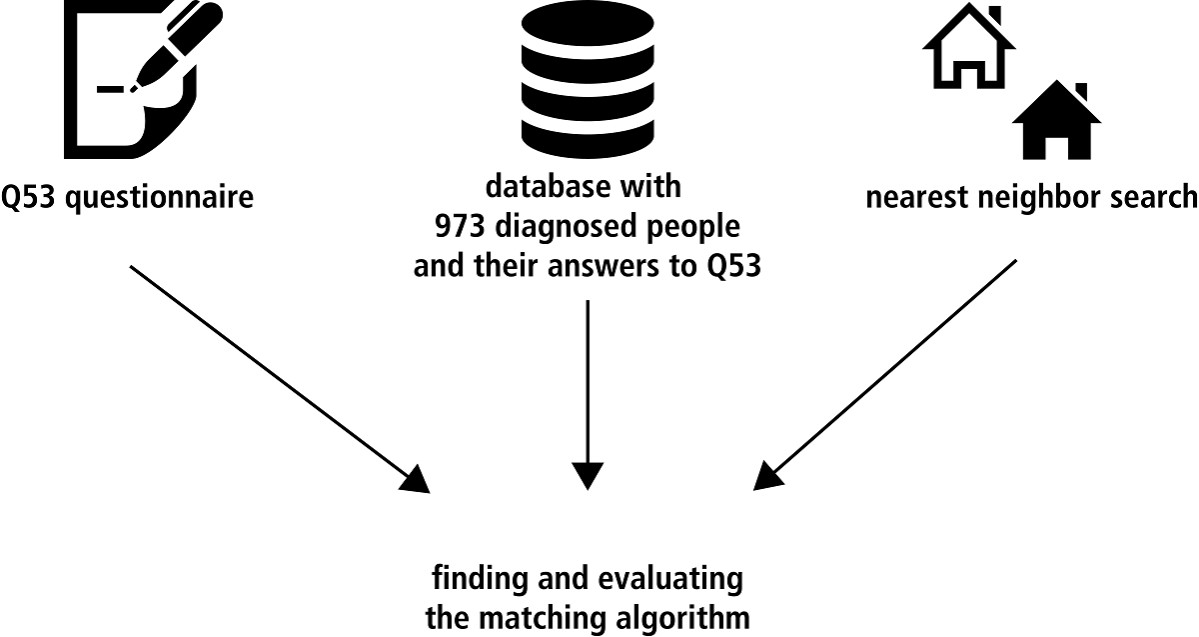
Used material for finding and evaluating the matching algorithm.

This basic process is illustrated in Figure 1. We used the k-nearest neighbor search and different distance calculating methods to find and evaluate matchings for a given set of 973 users with different diagnoses and different diagnostic categories. The k-nearest neighbor search is an easy, but effective, way to find similarities. Different calculating methods from different mathematical groups based on a publication by Cha [17] were compared.

In step 4, the prototype with the matching algorithm was evaluated based on leave-one-out cross-validation, which means that 1 data set was *left out* and the algorithm searched for fitting matches in the remaining 972 data sets. The matches for every single data set out of the total 973 answered Q53 questionnaires were analyzed regarding age, gender, latency (ie, *time with* symptoms but no diagnosis), disease group (category of the diagnosis referring to the affected organ or pathophysiology [eg, neuromuscular disease, metabolic disease]), diagnostic system (higher-order category that defines the type of the diagnosis [eg, RD or CD] but does not consider the affected organ [eg, RD of the liver]), and exact diagnosis (ie, exact name of the diagnosis). We considered matching for the same diagnosis in the category *diagnosis* most important, followed by the same *diagnostic system*, *disease group*, and *age*. The same *latency* and *gender* were considered less important for matching. Here, we followed the hypothesis that matching partners benefit most from sharing the same diagnoses or diagnostic categories. Such a matching might enable a helpful dialogue between the matching partners (eg, common experiences, doctors, therapies). By contrast, the same *gender* would not be as helpful. This analysis was performed for every calculating method, as well as a random sampling. Likewise, a comparison of random matching and similarity-based matching was possible.

## Results

### Overview of RarePairs User Path

**Figure 2:**
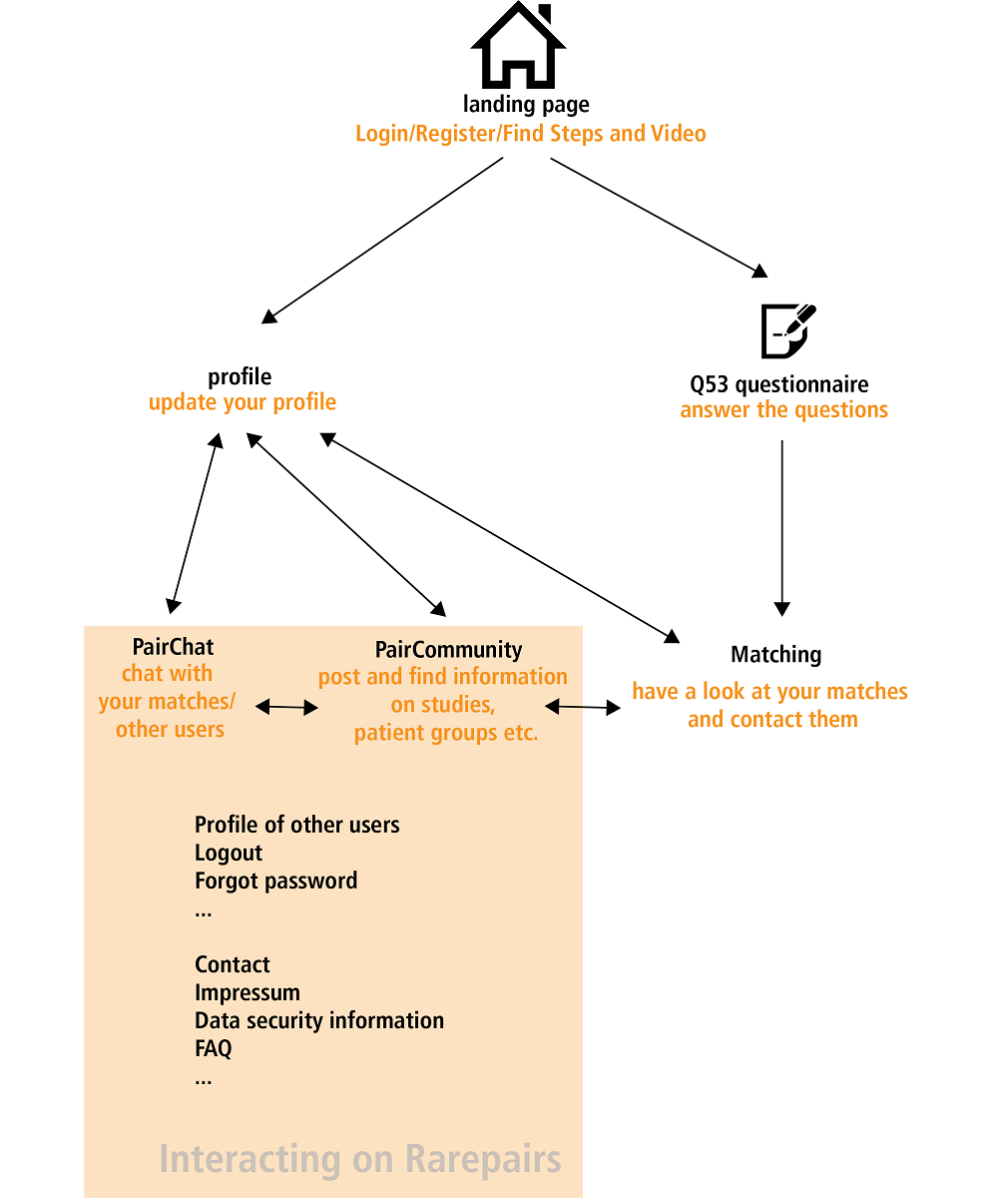
Simplified scheme of the clicking path for new and already registered users of RarePairs.

**Figure 3:**
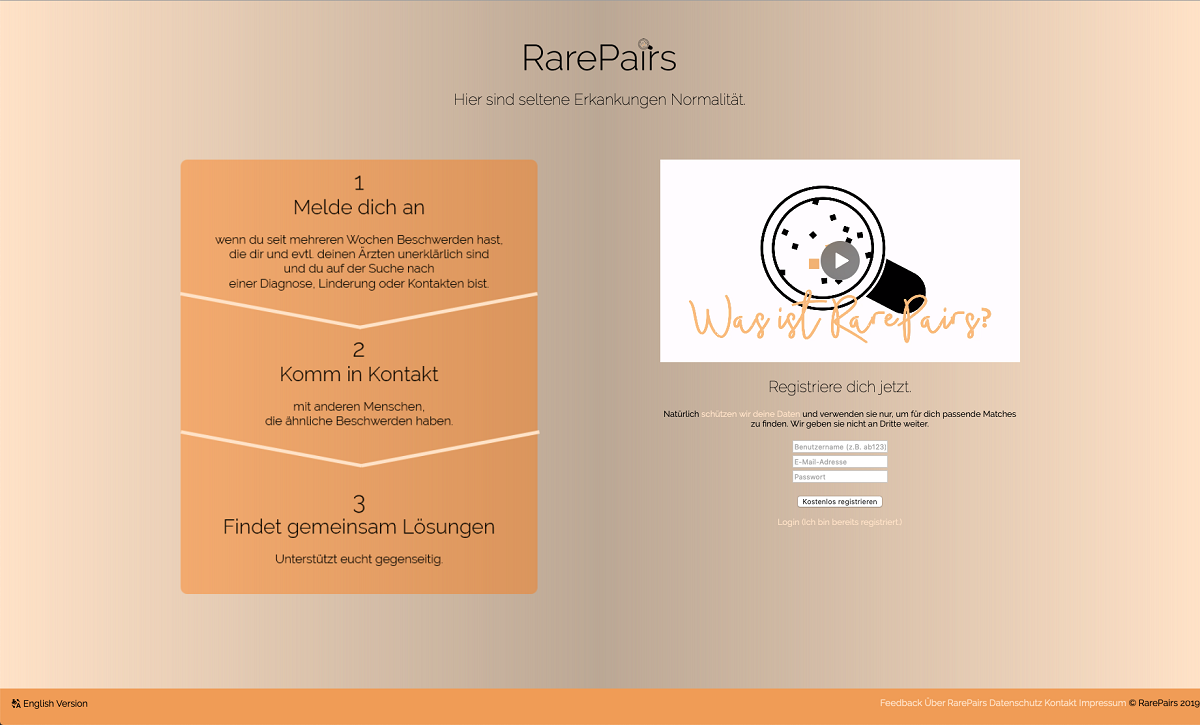
Landing page of RarePairs where users can get information, register, or log in. Users find information by text and a short video addressing aims and scope of RarePairs. Currently, the landing page is in German, an English version is under construction.

The users’ path through RarePairs is illustrated in Figure 2. After the login procedure, the user updates a profile and answers the 53 questions essential for matching (see Figure 2). The landing page of RarePairs is shown in Figure 3 (Further screenshots of the prototype can be found in Multimedia Appendix 4).

### Login/Register

Users register, or, if they already have an account, login into RarePairs. The usual basic security arrangements such as checking email format, checking password, transferring data as POST variables, not allowing multiple accounts with the same email address are made. Moreover, the users can request their password via email, if forgotten. The user data are stored in a MySQL database table titled *users*.

### Q53 Questionnaire

**Figure 4:**
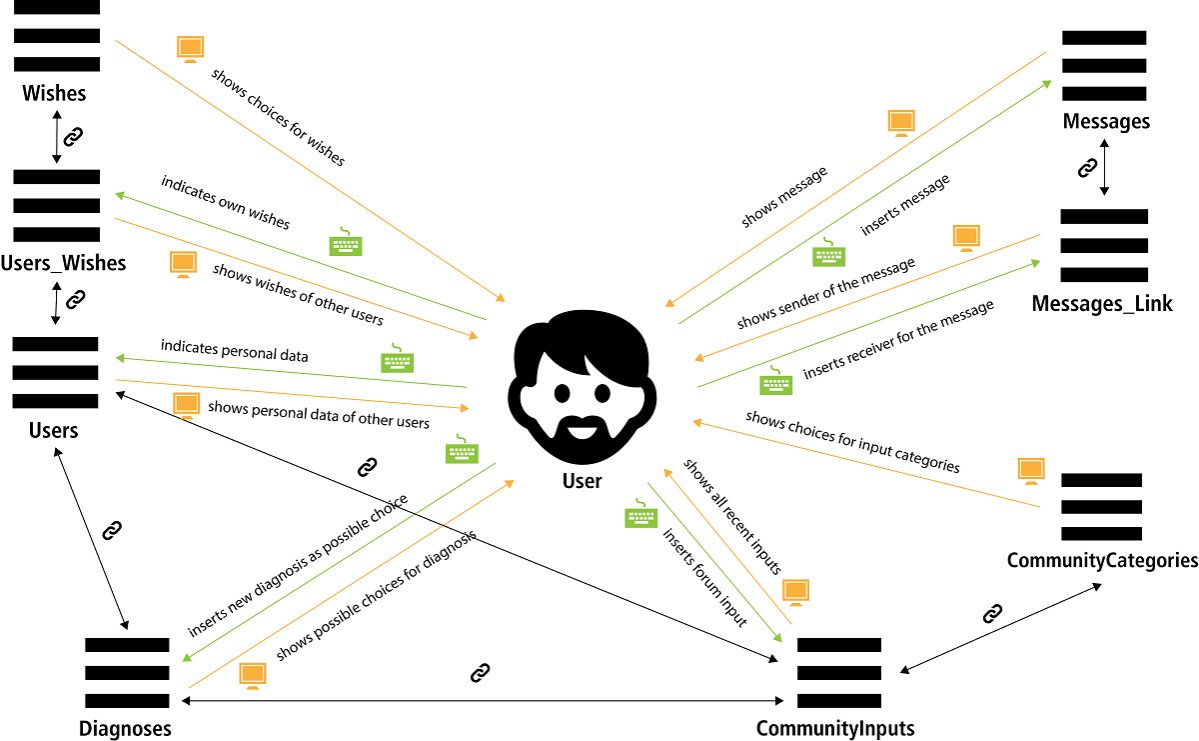
Visualization of the tables and possible interactions between them and the user.

New registering users are initially led to the first Q53 questionnaire sheet. The questionnaire is divided into 9 website sheets. The first explains the aims and scope of the Q53 questionnaire. Additionally, users have the opportunity to give supplementary information (eg, hobbies or the aims/wishes of the user) for the account profile. The in-between sheets (numbers 2-8) show the 53 questions and allow answering through PHP forms. Here, the data are transferred via POST variables and stored into the MySQL database table *users* (users aims/wishes are stored in the database ‘Wishes’ [table ‘Users_Wishes’]; see Figure 4 for all database tables and possible interactions). It is possible to change the personal profile data later. In the current prototypic version of RarePairs, the answers to the 53 questions of the Q53 questionnaire are static (ie, they cannot be changed later).

### Matching

The answer pattern of the Q53 questionnaire forms the basis for matching different users of RarePairs. In this prototype, the nearest neighbor was used to calculate matching users/similar answer patterns in the Q53 questionnaire.

Therefore, we calculated the differences of answers stored in the MySQL database table *users*. For calculating, many different distance (respectively similarity) calculating methods were compared such as Manhattan (d= Σ^d^_i=1_|P_i_ – Q_i_)|) or cosine (d=[Σ^d^_i=1_Pi·Qi]/[√Σ^d^_i=1_p^2^_i_·√Σ^d^_i=1_q^2^_i_]) from 8 different mathematical groups based on the publication by Cha [17]. As values, we used the numerical equivalents of given answers as explained above: (1) No, (2) Slightly no, (3) Slightly yes, (4) Yes, and (5) Do not know.

### Finding the Right Distance Calculating Method

**Figure 5:**
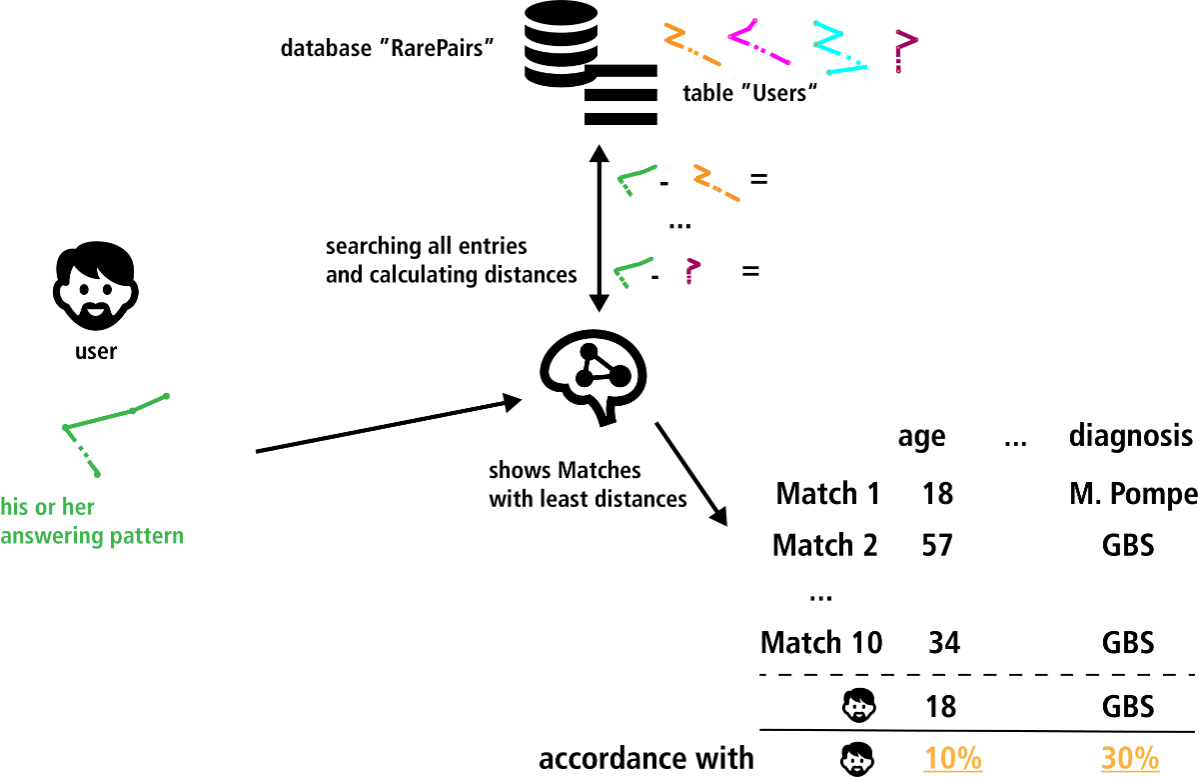
Illustration of the first part of identifying a matching method. Screening the data set for 10 "best" matches for one user (using the leave-one-out-method). This scheme only illustrates the basic principle of the simulation. The exact results are shown in Table 1. GBS: Guillain-Barré syndrome; M. Pompe: Morbus Pompe.

**Figure 6:**
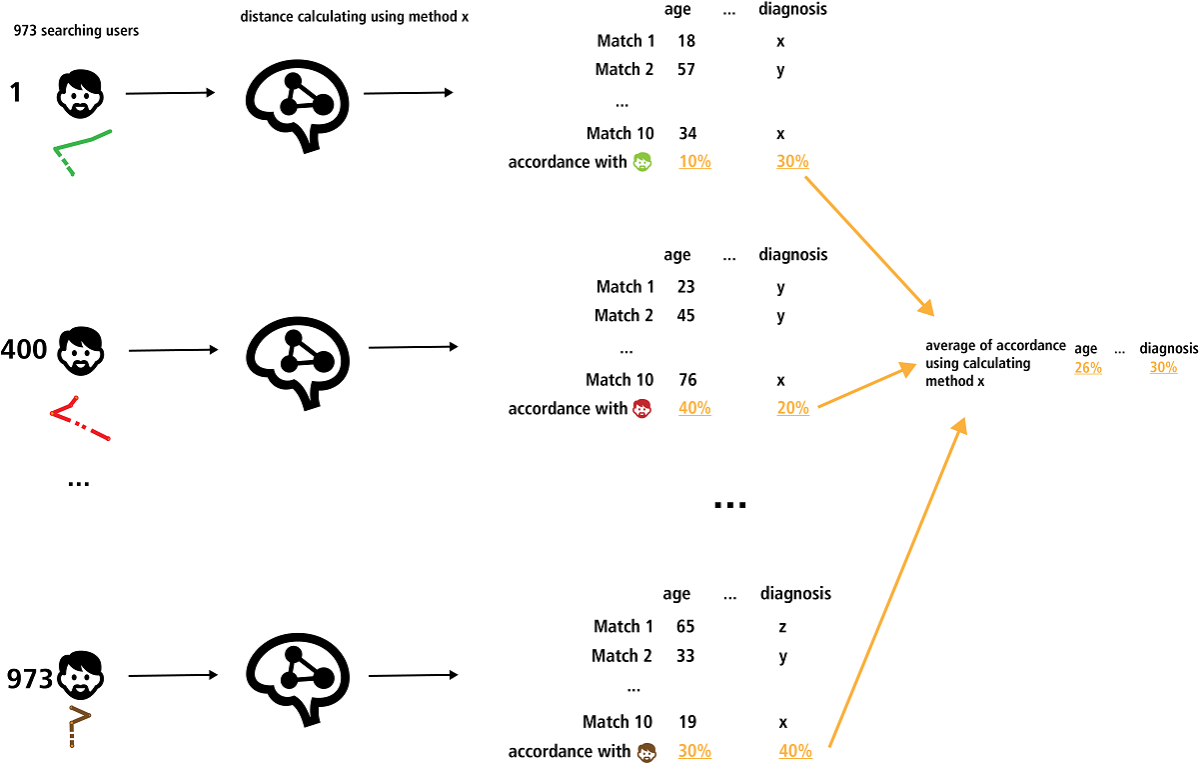
Schematic illustration of the second part: identifying a matching method for a given data set of users with rare diseases. Ten matches for all 973 data sets with one calculating method, calculating the average of the matching accordance of properties. This figure illustrates the basic principle of the simulation (for the complete testing results, see Multimedia Appendix 5 and Table 1).

From an AI perspective, it is not a priori clear as to which calculating method works best. Therefore, we compared matching with 24 different methods (see Multimedia Appendix 5 for the exact mathematics). We used the existing database of 973 data sets (containing personal information such as age and gender and all answers to the Q53 questionnaire), and identified 10 matches for every data set (using the leave-one-out method; see Figures 5 and 6 for visualization). We chose k=10 because we assumed it to be a good compromise between proper selection size for the searching user and still not be overwhelming. After finding matches with one calculating method, we repeated the process with another method. Second, we evaluated the matching by comparing the properties of the data set and its 10 matches, and the quality of the matching (eg, accordance of diagnosis = how many of the 10 matches have the same diagnosis as the user under evaluation). The average of the accordance for every calculating method is shown in Table 1 (this table is also added as Multimedia Appendix 6, with additional details). Comparisons of the average values indicate that only a few methods differ significantly (*P*>.05; see Table 1 for exact *P*-values).

**Table 1:** Different distance calculating methods simulated using the leave-one-out principle.

Distance calculating method		Matching accordance in percent													
		Gender^a^		Age^b^		Latency^c^		Disease group^d^		Diagnostic system^e^		Exact diagnosis^f^			
(Pseudo)random sampling (=negative benchmark)		59.6		15.7		11.9		8.3		31.7		3.8			
**L_p_ Minkowski family**															
	Manhattan		63.9^g^		21.9^g^		14.3		16.2^g^		40.3^g^		9.4^g^		
	Euclidean		62.4^g^		21.1		15.5^g^		16.0^g^		36.1^g^		9.7 (=positive benchmark)		
	Minkowski		63.8^g^		21.9^g^		14.4		16.2^g^		40.3^g^		9.4^g^		
**L_1_ family**															
	Sørensen		64.8^g^		22.1^g^		13.2		15.3^g^		37.8^g^		8.8		
	Gower		63.8^g^		21.9^g^		14.4		16.2^g^		40.3^g^		9.4^g^		
	Canberra		64.2^g^		21.5^g^		14.0		15.8^g^		37.7^g^		9.4^g^ (does not differ from the positive benchmark; *P*=.56)		
	Lorentzian		63.5^g^		21.7^g^		13.9		15.9^g^		39.4^g^		9.3^g^		
**Intersection family**															
	Wave Hedges		64.1^g^		21.5^g^		14.0		15.9^g^		38.3^g^		9.3		
	Czekanowski		64.8^g^		22.1^g^		13.2		15.3^g^		37.8^g^		8.8		
	Tanimoto		64.8 (=positive benchmark)		22.1 (=positive benchmark)		13.2 (differs from negative benchmark; *P*<.001)		15.3^g^		37.8^g^		8.8		
	Jaccard		63.8^g^		21.3		13.8		14.9		35.4^g^		8.7		
	Dice		63.8^g^		21.3 (differs from positive benchmark; *P*=.03)		13.8		14.9 (differs from positive benchmark; *P*=.05)		35.4^g^		8.7 (differs from negative benchmark; *P*<.001)		
**Inner product family**															
	Cosine		53.6^h^ (< negative benchmark)		12.0^h^ (< negative benchmark)		7.3^h^ (< negative benchmark)		4.7^h^ (< negative benchmark)		17.0^h^ (< negative benchmark)		1.9^h^ (< negative benchmark)		
**Fidelity family or Squared-chord family**															
	Bhattacharyya		64.8^g^		19.0 (differs from negative benchmark; *P*<.001)		7.9^h^ (< negative benchmark)		10.1^h^ (does not differ from negative benchmark; *P*=.70)		32.3 (differs from negative benchmark; *P*<.001; differs from positive benchmark; *P*<.001)		2.8^h^ (< negative benchmark)		
	Hellinger		62.9^g^		19.3		5.8^h^ (< negative benchmark)		7.4^h^ (< negative benchmark)		52.5 (=positive benchmark)		0.3^h^ (< negative benchmark)		
	Squared-Chord		62.0^g^		21.6^g^		15.3^g^		15.6^g^		35.2^g^		9.6^g^		
**Squared L_2_ family/dX^2^ family**															
	Neyman		59.5^h^ (< negative benchmark)		20.4		15.2 (differs from positive benchmark; *P*=.02)		14.9 (differs from negative benchmark; *P*<.001)		34.2^g^		9.0		
	Probabilistic Symmetric		62.2^g^		21.6^g^		15.1		15.8^g^		35.5^g^		9.7^g^		
	Clark		63.1^g^		21.4^g^ (does not differ from positive benchmark; *P*=.42)		14.8		15.3^g^		35.5^g^		9.3		
	Additive symmetric		61.3^g^ (does not differ from positive benchmark; *P*=.08)		21.1		15.7^g^		15.5^g^		34.1^g^		9.5^g^		
**Shannon’s entropy family**															
	Jeffreys		62.0^g^		21.5^g^		15.3^g^		15.6^g^		35.1^g^		9.6^g^		
	Jensen difference		62.1^g^		21.5^g^		15.3^g^ (does not differ from positive benchmark; *P*=.55)		15.7^g^		35.3^g^		9.6^g^		
**Combined methods**															
	Kumar–Johnson		60.8 (differs from positive benchmark; *P*=.02)		21.2		15.7 (=positive benchmark)		15.0^g^ (does not differ from positive benchmark; *P*=.08)		33.4^g^ (does not differ from positive benchmark; *P*=.14)		9.3^g^ (differs from positive benchmark; *P*=.02)		
	Avg		63.7		21.8^h^		14.4		16.4 (=positive benchmark)		40.3^g^		9.4^g^		

^a^Gender of the person.

^b^Age of the person

^c^Time with symptoms but no diagnosis.

^d^Category of the diagnosis referring to the affected organ or pathophysiology (eg, neuromuscular disease, metabolic disease)

^e^Greater category the diagnosis can be assigned to (eg, RD, CD), not especially considering the affected organ

^f^Exact name of the one diagnosis.

^g^Fields do not differ significantly from the positive benchmark in this category; see *P*-value in those fields. If no *P-*values are mentioned, the matching values lie in between the benchmark and the furthest value, which is only just not differing significantly from this benchmark.

^h^Fields do not differ significantly from the (pseudo)random matching (=negative benchmark); see *P*-value in those fields. If no *P*-values are mentioned, the matching values lie in between the benchmark and the furthest value, which is only just not differing significantly from this benchmark.

**Figure 7:**
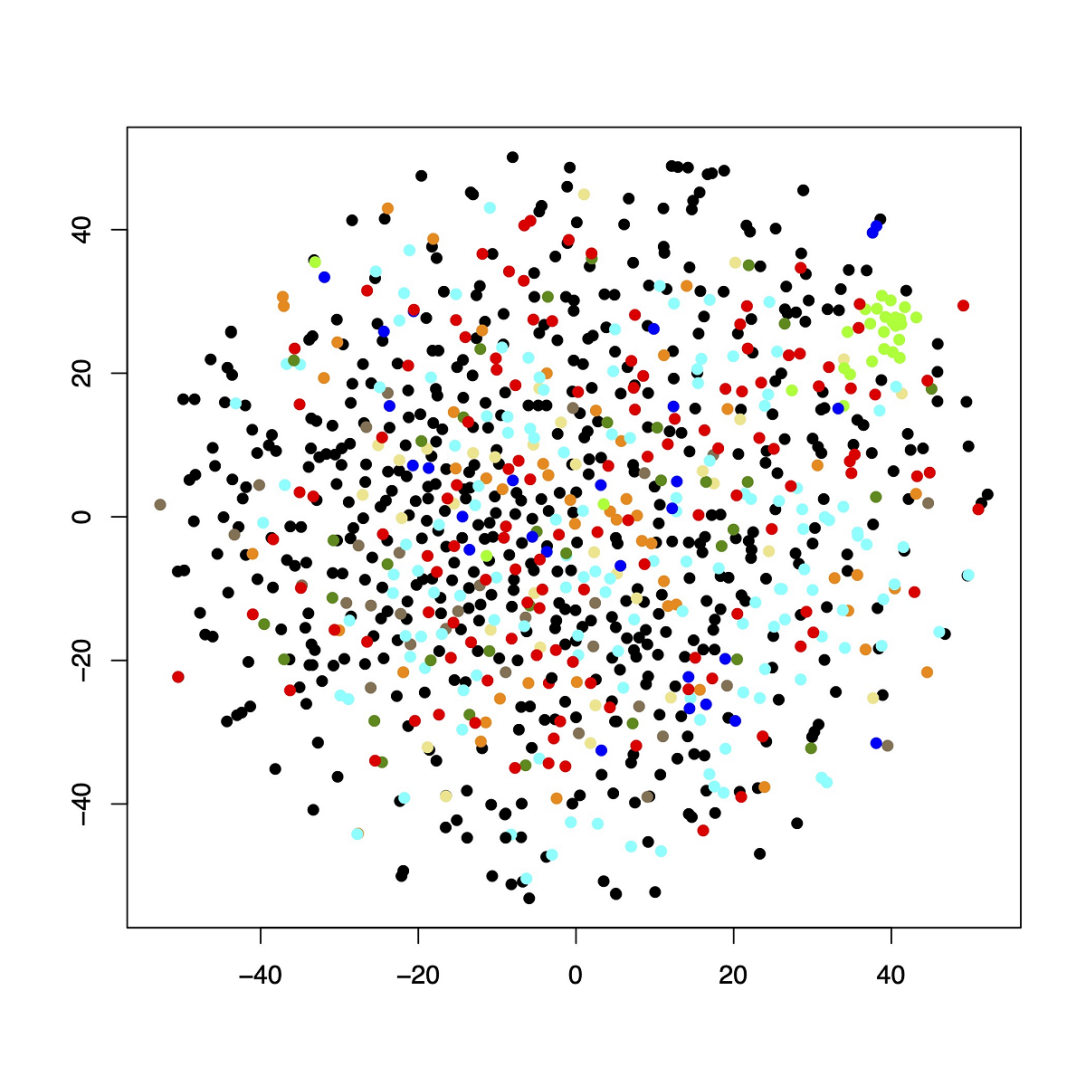
A t-distributed stochastic neighbor embedding plot showing a possible clustering of the 973 test objects concerning the diagnostic system of their disease. Key: black: rare diseases; red: chronic diseases; dark blue: psychiatric diseases with somatoform part; dark green: unknown diagnosis; light green: healthy individuals; light blue: sarcoidosis; orange: idiopathic pulmonary arterial hypertonia; yellow: syringomyelia; brown: systemic lupus erythematosus.

Furthermore, we calculated the average values for a random matching and comparison, showing that most of the calculating methods resulted in significantly better results than the random matching (*P*≤.05; see Table 1 for exact *P*-values). These results support our assumption that the k-nearest neighbor search itself is a robust base for the matching algorithm. Additionally, the cosine method, which mathematically produces matches of people with preferably different answers, showed the unfavorable results as expected. To make sure the results fit the outcome that could be expected from the 973 people data set, we plotted the 973 people by the diagnostic system of their disease using the t-distributed stochastic neighbor embedding method. The plot (Figure 7) illustrates that there is no clustering except for the group of healthy individuals (green dots). This finding underlines that the nearest neighbor method would produce poor results when used (solely) for classification. In RarePairs, we used this method to find suitable matches (and not to classify our data). The results in Table 1 illustrate that the nearest neighbor search is suitable for matching users answering the 53-item questionnaire under discussion in this study. In this set of data, the Jeffreys, squared-chord, or Jensen difference method proved most powerful.

### Interacting on RarePairs

Once the user has found matches, s/he can interact with his/her new matches as well as other users. We implemented a few basic methods to illustrate this function.

#### PairChat

For interacting with 1 single user, a given user can use the *PairChat* function. PairChat is programmed as a common chat tool with the obvious functionalities of writing and receiving messages and reading them in a messenger window. Written messages are stored in the database table called *Messages*, and the link between messages and their receiver and sender is stored in the table *Messages_Link*. For showing all the messages that were exchanged between 2 users, the PHP script looks for all messages in *Messages_Link* that were sent or received by the active user, and shows them sorted by the corresponding sending or receiving chat partner and date/time.

#### PairCommunity

We designed the *PairCommunity* as a *broadcast forum* for all RarePairs users. Here they can share important information that might interest all registered users such as invitation to self-help groups or announcements about current scientific events. It is possible to sort the posts by different categories (database table *CommunityCategories*), such as self-help-groups or leisure time groups. The posts themselves are stored in the database table *CommunityInputs.*

## Discussion

### Principal Findings

In this study we outlined, created, and evaluated a prototype of a social media platform (RarePairs) for individuals with (undiagnosed) RD conditions. Evaluation of RarePairs using a single statistic function on an existing set of data illustrated decent matching results for possible users searching for a diagnosis.

About 35% of Americans use the internet for finding a diagnosis [18] and over half the global population use social media [19]. Hence, the idea of creating an online social network for people looking for a diagnosis seems to be a logical step. In that context, Russell et al [20] installed and observed a Facebook group where parents of disabled children and researchers were linked together and discussed medical studies, everyday struggles, and disease challenges. According to their analysis, 95% of the parents were motivated to join the group for connecting with like-minded people, 78% were using the group to find information, and 73% wanted to receive or give emotional support. Although the focus of that project was more in the context of research and understanding the use of social media in the context of a given medical context, the results indicate that users appreciate beneficial effects of social media in certain medical or medicosocial contexts. A popular example of a diagnostic-support online platform is *CrowdMed,* where professionals and nonprofessionals can engage in trying to help undiagnosed individuals find the right diagnosis. In contrast to our project, where diagnostic support is based on using a questionnaire, in CrowdMed patients share medical information (eg, medical reports, laboratory data). Meyer et al [21] reported first successes for a few participants and 56.9% of participants reported that the hints given by others on CrowdMed led them closer to the right diagnosis [21].

A common motivation brings individuals with various diagnoses together in *real life* self-help groups. Plinsinga et al [22] performed a survey on individuals with osteoarthritis and their interest in self-help-groups [22]. In that study, 307 of the 415 included patients were interested in participating in a self-help group; 54% of the patients reported to be engaged in a patient group, whereas 41% reported participation in an online self-help group (namely through Facebook). Such data illustrate the tremendous motivation among individuals with CD or RD to connect and support each other. RarePairs fills in a gap between individuals without diagnosis and those knowing the name of their RD.

Establishing an online social network for undiagnosed people seems to be an opportunity for younger adults during their diagnostic odyssey. They are even more likely to search for help on the internet and are more accustomed to using the internet. Lee et al [23] stated that people aged 55 or older have more difficulties finding the right (health) information on the internet than younger people.

Especially in the context of RDs, a social platform as illustrated in our study seems beneficial. Here, affected individuals from different countries could be easily connected and inspired to exchange valuable health care information crossing borders and even continents. Such a technology might be especially valuable for ultra-RD conditions with only a handful of affected individuals worldwide.

Individuals with experience in using social media and performing diagnostic research on the internet might find a platform like RarePairs advantageous. However, there might be criticism that *Googling* symptoms results in wrong information. Concerns that patients may have trouble following the doctor’s recommendations after reading online information prove mostly wrong [24], and such well-informed (via the internet) patients may even help the doctor with the diagnosis [25]. Besides the opportunity of *Googling* symptoms, there is also a lack of reliable online information [26]. Moreover, wrong or disturbing information from the web might disturb the patient–doctor relationship or produce *cyberchondriacs* [27,28].

Of course, there are indicators that the internet will be a growing source of diagnostic help, but there is a lack of well-designed online tools and websites with relevant and quality-proven information [26]. With RarePairs we address those needs and promote a completely different strategy: by using a simple, but powerful questionnaire (Q53), users are connected without needing profound medical knowledge. In the future, this questionnaire-based tool might be improved using additional information from, for example, wearables. Additionally, the previous study [16] used a combination of different AI methods (support vector machine, random forest, logistic regression, and linear discriminant analysis), with better results in clustering diseases [16]. Perhaps the use of those additional AI methods could also improve the matching algorithm of RarePairs.

Analyzing the matching algorithm was an important result of this study, highlighting that nearest neighbor methods worked significantly better than a random matching. For improving the test results, there is still room for improvements by including and evaluating other AI methods in combination with the nearest neighbor search in future research projects.

Today, there are almost daily new highlights addressing improvements in the field of diagnostic support through AI (the number of publications on PubMed containing the words AI are 10 times as high as in the 1990s, as per our manual search on the database in 2020). The fields where such algorithms already are in use are mainly within visual diagnostics (radiology, pathology, dermatology, microbiology). For example, support for doctors is tested during a polyp screening [29], predicting a coronary artery disease noninvasively, or diagnosing a sepsis in an early state [30,31]. Nevertheless, in everyday clinical use, there are also challenges such as server infrastructure or computer power that have to be overcome [32].

Data security of (disease-specific) personal data is an important issue. In RarePairs the stored data are protected by an SSL connection and cannot be linked to an address or even a name, and as such there is a guarantee for basic data safety for all users. Encrypting the stored data could be a next step for the time RarePairs will be used as a real marketable product. Also building a decentralized database could be a very elegant way to make the user data safer during the next steps of development.

### Limitations

The complete realization of RarePairs is currently only prototypic. Accordingly, there are several aspects about RarePairs that have to be addressed in the future: We set the user requirements and context of use based on information and selected assumptions. In future studies, the user satisfaction needs evaluation. Besides, it is essential to test the prototype with a larger target audience in the future and reevaluate the results.

A second limitation is the small database used for this prototype. These data might not automatically reflect the community of possible users of such a network. Consequently, the results can only be regarded as a milestone. Additionally, all records of this study belonged to individuals having their diagnosis fixed. The prototype was not tested with real individuals during their *diagnostic journey*. An evaluation with more diverse data will be performed during the next steps of RarePairs’ development. Furthermore, one could question the quality criteria of a *perfect match* (same gender, same age, same diagnosis) because we do not know which persons would profit from each other *in reality*. That is why we suggest planning a prototype test phase, and until then ask the participating persons to evaluate the quality of the suggested matches. Constant adjustment of the matching methods will be part of RarePairs while in daily use.

Another limitation of this analysis is that we only evaluated the data set and matching quality by just randomized matching. One might criticize that such an evaluation is only a low bar challenge. Assuming that the user perspective on the matching quality is completely unknown, we decided that this testing fulfills the evaluation of a prototype. Further steps for evaluating the algorithm have to follow.

Additionally, as the Q53 questionnaire was designed and developed from a German perspective, its success and performance must be re-evaluated in different cultural contexts. Today, translated versions are available in English, Chinese, Portuguese, and Finnish, but a systematic trans-cultural evaluation was not yet performed.

From the technical perspective, the current prototype is restricted to usage on a personal computer (and therefore we did not use a CSS Template). We are well aware that in the context of constant growth and increasing numbers of RarePairs users, adoption of the code, and focus on the development of a cross-platform app/website, as most people prefer to use social media on their mobile device [33], a template would be useful. This prototypic evaluation was only designed to be a scientific *proof of concept* and therefore uses an easy-to-use and flexible programming technique. The development of a mobile app is the next logical step.

For the use of forms, JavaScript could also be of advantage because it makes the forms more interactive and the entries can be checked more easily. Concerning the details of the prototype, there are a few functionalities that should be implemented in the future:

- Answers to the Q53 questionnaire should be changeable if the user gets new knowledge about their disease, or if the experience of the symptoms/disease changes. There must be a possibility to get new matches based on these changed answers.
- The display of the matches should contain a form of ranking, possibly showing how many questions were answered similarly or which questions had the biggest effect for the matching (that is how the diagnostic app *Ada* [34] does it.).
- It should be possible to search for other users even if they are not a fitting match (eg, to find friends from real life).
- The *PairCommunity* should be searchable for posts concerning different diagnoses.
- A contact form should be implemented.
- The chat could be using the XMPP (Extensible Messaging and Presence Protocol) to make cross-platform chatting possible.

Besides, any tool in the context of diagnostic support must obviously prevent individuals in despair from raising hope for easy solutions or diagnoses using the internet or a given platform. This cannot be offered by RarePairs in its current structure, and consequently is only one piece in a larger puzzle to support individuals during a difficult diagnostic search. RarePairs therefore strongly underlines that it is not designed as a diagnostic tool (eg, via disclaimers during the registering process).

### Next steps

Our next goal is to present RarePairs to real users (eg, in self-help groups) and collect feedback systematically. For a first round with approximately 500 users, we would need additional resources for legal advice, implementing new functions and providing more (data) security.

## Appendix

Multimedia Appendix 1 Analytic steps for the preparation of RarePairs.

Multimedia Appendix 2 Questionnaire Q53 [german].

Multimedia Appendix 3 Questionnaire Q53 [english].

Multimedia Appendix 4 Collection of RarePairs Screenshots.

Multimedia Appendix 5 Methods for distance calculating.

Multimedia Appendix 6 Different distance calculating methods simulated using the leave-one-out-principle.

## Abbreviations

AI: artificial intelligence
CD: chronic disease
EU: European Union
RD: rare disease
