# Supplementary material for: Development of a Social Network for People Without a Diagnosis (RarePairs): Evaluation Study
Source: J Med Internet Res. 2020 Sep 29;22(9):e21849. doi: 10.2196/21849 (PMC7556379; doi:10.2196/21849)
Supplement: Multimedia Appendix 1 [file jmir_v22i9e21849_app1.docx]

Table: Analytic steps for the preparation of RarePairs

| **Step** | **Result** |
| --- | --- |
| 1. systematic literature research | overview on current concepts and limitations in the field |
| 1. contact with patient groups | naming of deficits for individuals |
| 1. contact with scientific focus groups | naming of needs for a social media platform |
| 1. contact with experts for RD and their diagnostic odyssey | designation of needs for a social media platform |
