# Supplementary material for: Development of a Social Network for People Without a Diagnosis (RarePairs): Evaluation Study
Source: J Med Internet Res. 2020 Sep 29;22(9):e21849. doi: 10.2196/21849 (PMC7556379; doi:10.2196/21849)
Supplement: Multimedia Appendix 2 [file jmir_v22i9e21849_app2.pdf]

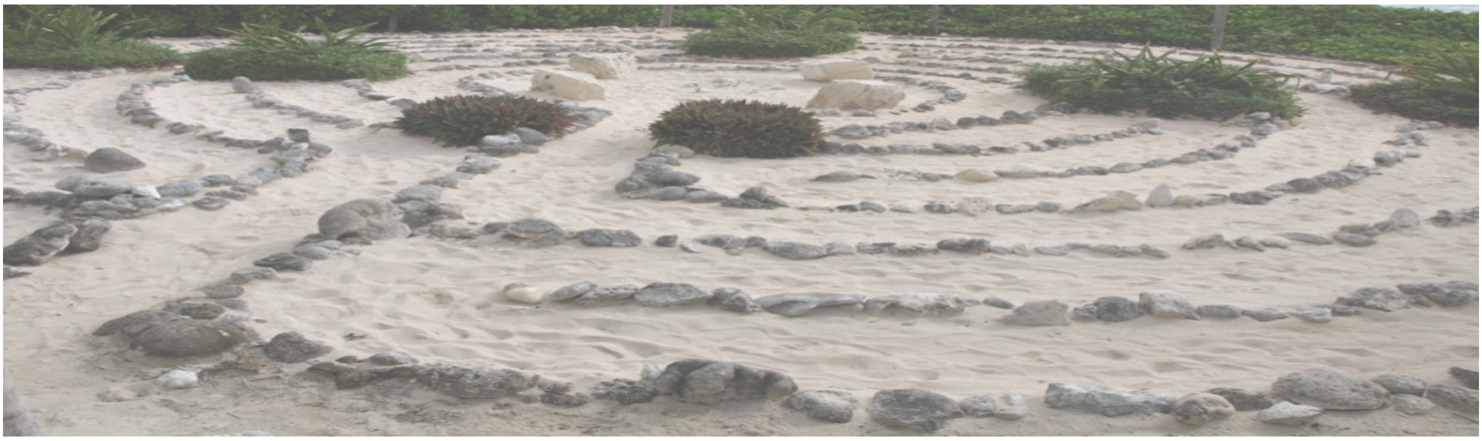

Sie haben eine Seltene Erkrankung? Sie sind krank – aber niemand findet die Ursache? Es geht Ihnen schlecht – aber niemand glaubt Ihnen? Sie leiden an einer chronischen Erkrankung?

IHRE ERFAHRUNG ZÄHLT!

Sehr geehrte Damen und Herren,

das Projekt *Kurze Wege zur Diagnose* hat sich zum Ziel gesetzt, Menschen mit Hilfe eines Fragebogens und spezieller Computertechnik schneller zur Diagnose zu führen. Der beiliegende Fragenkatalog ist das Ergebnis der Auswertungen von Interviews mit erwachsenen Betroffenen sowie mit Eltern betroffener Kinder.

Nun benötigen wir auch ***Ihre Hilfe*** und ***Ihr Erfahrungswissen***. Wir möchten Sie höflich bitten, den nachfolgenden Fragebogen auszufüllen und sich hierfür in **die Zeit, bevor Ihre Beschwerden einen Namen *(eine „Diagnose") hatten*, zurückzuversetzen.**

Die Beantwortung der Fragen wird nur einige Minuten dauern. Alle Angaben werden **komplett anonymisiert** und ausschließlich für das geschilderte Projekt verwendet.

Für Fragen, Rückmeldungen und Anregungen stehen wir Ihnen jederzeit sehr gerne zur Verfügung.

Vielen Dank für Ihre Mithilfe!

PD Dr. Lorenz Grigull, Susanne Blöß (B.A.), Sandra Mehmecke (M.A.)

EINE BEFRAGUNG DER:

MHH

im Rahmen des Projekts

Seltene chronische Erkrankungen – Kurze Wege zur Diagnose

KONTAKT:

Medizinische Hochschule Hannover
Abteilung Päd. Hämatologie und Onkologie
Carl-Neuberg-Straße 1
30625 Hannover

E-Mail: **Bloess.Susanne@mh-hannover.de**
Tel: **0511-532-9476**
Fax: 0511-532-16-9467
Homepage: https://www.mh-hannover.de/kurzewege.html

GEFÖRDERT VON:

Robert Bosch Stiftung

Den Fragebogen finden Sie auch online unter: http://imdiagnostics.com/selten
mit dem Passwort: „selten"

Im Folgenden finden Sie eine Reihe von Fragen. Als Antwortmöglichkeiten können Sie für jede Frage zwischen **„nein“**, **„eher nicht“**, **„eher schon**“, **„ja“** und **„weiß nicht“** wählen. Bitte machen Sie entsprechend Ihrer Antwort **jeweils nur ein Kreuz**. Dabei können Sie gar nichts falsch machen, denn es gibt keine für jede Person zutreffenden Antworten.

I. ALLGEMEIN:

Diagnose (falls bekannt):

Sie beantworten den Fragebogen als Betroffene(r) ☐ Sie beantworten den Fragebogen als Angehörige(r) ☐

Geschlecht: weiblich ☐ männlich ☐

Alter in Jahren: Dauer Ihrer Diagnosesuche in Jahren:

Fragebogen-ID (bitte nicht ausfüllen):

II. URSACHENSUCHE:

	Antwortmöglichkeiten:	nein	eher nicht	eher schon	ja	weiß nicht
**1.**	Ahnen/ ahnten Sie – ggf. schon länger – dass „etwas“ mit Ihnen **nicht** stimmt?	☐	☐	☐	☐	☐
**2.**	Trifft es zu, dass Ihre Beschwerden/ irritierenden Erscheinungen bei Ärzten/ Ihrem Arzt erst einmal ohne Reaktion blieb?	☐	☐	☐	☐	☐
**3.**	Hat man bei Ihnen auffällige Untersuchungsergebnisse *(z.B. Blutwerte, EKG, Hautveränderungen, usw.)* festgestellt, deren Ursache jedoch zunächst **nicht** weiter untersucht wurde?	☐	☐	☐	☐	☐
**4.**	Trifft es zu, dass es schwierig für Sie ist/ war, Ihre Beschwerden/ irritierenden Erscheinungen in Worte zu fassen?	☐	☐	☐	☐	☐
**5.**	Haben Sie es jemals für möglich gehalten, dass die Ursache für Ihre Beschwerden/ irritierenden Erscheinungen in Ihren Lebensgewohnheiten *(z.B. Ernährung, Wohnverhältnisse, Reisen, usw.)* liegen könnte?	☐	☐	☐	☐	☐
**6.**	Wurden bei Ihnen viele Untersuchungen durchgeführt, die ohne Ergebnis blieben?	☐	☐	☐	☐	☐
**7.**	Gingen oder gehen Sie immer wieder mit denselben Beschwerden/ irritierenden Erscheinungen zu Ärzten/ Ihrem Arzt?	☐	☐	☐	☐	☐
**8.**	Haben Sie wegen unterschiedlicher Beschwerden/ irritierender Erscheinungen verschiedene Fachärzte aufgesucht?	☐	☐	☐	☐	☐
**9.**	Erinnern Sie sich – im Zusammenhang mit Ihren Beschwerden – an eine Situation, die Sie als besonders bedrohlich erlebt haben?	☐	☐	☐	☐	☐
**10.**	Setzen Sie im Alltag spezielle Techniken *(Tricks und Kniffe)* ein, um Ihre Einschränkungen auszugleichen?	☐	☐	☐	☐	☐
**11.**	Vermeiden Sie bewusst Aktivitäten (Tätigkeiten), bei denen Ihre Beschwerden/ irritierenden Erscheinungen sichtbar werden?	☐	☐	☐	☐	☐
**12.**	Haben Sie sich aus eigener Initiative heraus über mögliche Ursachen Ihrer Beschwerden/ irritierenden Erscheinungen informiert?	☐	☐	☐	☐	☐
**13.**	Haben Ihre Beschwerden/ irritierenden Erscheinungen im Laufe der Zeit immer wieder andere Namen (Diagnosen) erhalten?	☐	☐	☐	☐	☐
**14.**	Haben Sie im Verlauf Ihrer Diagnosesuche von sich aus nach Ärzten (Spezialisten, Experten) gesucht?	☐	☐	☐	☐	☐
**15.**	Haben Sie schon einmal die Durchführung von **Untersuchungen** von Ärzten/ Ihrem Arzt gefordert?	☐	☐	☐	☐	☐
**16.**	Haben Sie den Eindruck, dass die von Ihnen geschilderten Beschwerden von Ärzten/ Ihrem Arzt ernst genommen werden/ wurden?	☐	☐	☐	☐	☐

	Antwortmöglichkeiten:	nein	eher nicht	eher schon	ja	weiß nicht
**17.**	Trifft es zu, dass Sie Ihr Vertrauen in Ärzte/ Ihren Arzt verloren haben?	☐	☐	☐	☐	☐
**18.**	Hatten Sie schon einmal den Punkt erreicht, an dem Sie Ihre Diagnosesuche aufgegeben haben?	☐	☐	☐	☐	☐
**19.**	Trifft es zu, dass bei Ihnen eine psychische/ psychosomatische Erkrankung *(z.B. aufgrund von Stress, traumatischen Erlebnissen, usw.)* vermutet wird/ wurde?	☐	☐	☐	☐	☐
**20.**	Gab es ein bestimmtes Erlebnis, dass Ihnen die Zunahme *(Verschlechterung)* Ihrer Beschwerden besonders vor Augen führte?	☐	☐	☐	☐	☐

III. KRANKHEITSZEICHEN:

	Antwortmöglichkeiten:	nein	eher nicht	eher schon	ja	weiß nicht
**21.**	Haben/ hatten Sie häufig erhöhte Temperatur (Fieber)?	☐	☐	☐	☐	☐
**22.**	Leiden Sie an mehreren Beschwerden/ irritierenden Erscheinungen zugleich *(z.B. Husten* ***und*** *Gewichtsverlust / Hautausschlag* ***und*** *Leistungsknick* ***und*** *Sehverschlechterung)*?	☐	☐	☐	☐	☐
**23.**	Leiden Sie unter ständiger Müdigkeit?	☐	☐	☐	☐	☐
**24.**	Haben/ hatten Sie eine irritierende Besonderheit *(z.B. Verfärbungen der Haut, Größerwerden von Körperteilen, Zittern, Zuckungen, usw.)* an sich festgestellt?	☐	☐	☐	☐	☐
**25.**	Leiden/ litten Sie immer wieder unter starke Schmerzen?	☐	☐	☐	☐	☐
**26.**	Hat Ihre körperliche Leistungsfähigkeit *(z.B. im Sport, beim Treppensteigen, usw.)* spürbar abgenommen?	☐	☐	☐	☐	☐
**27.**	Haben Sie Atembeschwerden an sich festgestellt *(z.B. Kurzatmigkeit bei Belastung, starker Husten, Schnarchen, Atemaussetzer, usw.)*?	☐	☐	☐	☐	☐
**28.**	Trifft es zu, dass Sie einen unsicheren Gang haben *(z.B. weil Sie schwanken, stolpern, stürzen, usw.)*?	☐	☐	☐	☐	☐
**29.**	Waren Sie aufgrund Ihrer Beschwerden schon einmal voller Verzweiflung?	☐	☐	☐	☐	☐
**30.**	Trifft es zu, dass Sie von Menschen aus Ihrem Umfeld *(Familie, Bekannte, Freunde, Kollegen, usw.)* auf körperliche Auffälligkeiten angesprochen wurden?	☐	☐	☐	☐	☐

IV. BESCHWERDEN UNTER KONTROLLE BRINGEN:

	Antwortmöglichkeiten:	nein	eher nicht	eher schon	ja	weiß nicht
**31.**	Haben Sie mit der Zeit gelernt, Ihre Beschwerden besser einzuschätzen?	☐	☐	☐	☐	☐
**32.**	Haben Sie von sich aus versucht, Ihre Beschwerden zu lindern *(z.B. durch Schmerzmittel, Salben, Wickel, Sport, besondere Ernährung, usw.)*?	☐	☐	☐	☐	☐
**33.**	Haben Sie bei Ärzten/ bei Ihrem Arzt schon einmal auf eine bestimmte **Behandlung (Therapie)** bestanden?	☐	☐	☐	☐	☐

V. BESONDERS SEIN:

	Antwortmöglichkeiten:	nein	eher nicht	eher schon	ja	weiß nicht
**34.**	Trifft es zu, dass Sie Dinge können, die Andere nicht können – oder anders herum?	☐	☐	☐	☐	☐
**35.**	Werden Ihnen Ihre Beschwerden/ irritierenden Erscheinungen erst im Vergleich mit „gesunden“ Menschen deutlich?	☐	☐	☐	☐	☐

	Antwortmöglichkeiten:	nein	eher nicht	eher schon	ja	weiß nicht
**36.**	Galten Sie als Kind/ im jugendlichen Alter als unsportlich *(waren Sie z.B. vom Schulsport befreit oder nahmen hieran ungern teil)*?	☐	☐	☐	☐	☐
**37.**	Stehen/ standen Sie aufgrund Ihrer Beschwerden/ irritierenden Erscheinungen häufig im Mittelpunkt?	☐	☐	☐	☐	☐
**38.**	Trifft es zu, dass Sie sich für sichtbare Veränderungen schämen/ schämten?	☐	☐	☐	☐	☐
**39.**	Sind Ihre Beschwerden für Andere verborgen bzw. äußerlich unsichtbar?	☐	☐	☐	☐	☐
**40.**	Haben Sie den Eindruck, dass andere Menschen auf Sie Rücksicht nehmen müssen?	☐	☐	☐	☐	☐
**41.**	Haben/ hatten Sie Ihre Beschwerden so weit wie möglich zurückgedrängt, im Bemühen, ein normales Leben zu führen?	☐	☐	☐	☐	☐
**42.**	Ist/ war die Ungewissheit über die Ursache Ihrer Beschwerden für Sie das Schlimmste?	☐	☐	☐	☐	☐
**43.**	Trifft es zu, dass Sie schon einmal selbst geglaubt haben, sich Ihre Beschwerden nur einzubilden (einzureden)?	☐	☐	☐	☐	☐

VI. SOZIALES UMFELD:

	Antwortmöglichkeiten:	nein	eher nicht	eher schon	ja	weiß nicht
**44.**	Haben/ hatten Sie den Eindruck, dass Ihr Umfeld *(Familie, Freunde, Bekannte, Kollegen, usw.)* Ihre Beschwerden **nicht** ernst nimmt *(z.B. dass jemand sagt: „Ist doch nicht so schlimm…“)*?	☐	☐	☐	☐	☐
**45.**	Trifft es zu, dass Sie Ihrem Umfeld *(z.B. Familie, Bekannte, Freunde, Kollegen)* Informationen zu Ihren Beschwerden/ irritierenden Erscheinungen bewusst vorenthalten?	☐	☐	☐	☐	☐
**46.**	Trifft es zu, dass Sie lieber zu Hause bleiben *(z.B. weniger mit Freunden unternehmen, usw.)*, seit Ihre Beschwerden/ irritierende Erscheinungen deutlich sind?	☐	☐	☐	☐	☐
**47.**	Haben Sie eine bestimmte Vertrauensperson, auf die Sie sich – im Zusammenhang mit Ihren Beschwerden/ irritierenden Erscheinungen – zu 100% verlassen können?	☐	☐	☐	☐	☐

VII. ALLTAG:

	Antwortmöglichkeiten:	nein	eher nicht	eher schon	ja	weiß nicht
**48.**	Verzichten Sie auf bestimmte Aktivitäten, die Ihnen eigentlich Freude machen?	☐	☐	☐	☐	☐
**49.**	Haben Sie aufgrund Ihrer Beschwerden/ irritierender Erscheinungen Veränderungen in Ihrem Alltag (*z.B. Tagesablauf geändert, alternative Wege gewählt*) vorgenommen?	☐	☐	☐	☐	☐
**50.**	Trifft es zu, dass Sie Unternehmungen *(z.B. Ausgehen, Tagesausflüge, Urlaubsreisen, usw.)* sehr vorrausschauend und genau planen/ planten?	☐	☐	☐	☐	☐
**51.**	Greifen Sie auf Hilfsmittel zurück, um im Alltag besser zurecht zu kommen?	☐	☐	☐	☐	☐
**52.**	Ziehen Sie aufgrund Ihrer gesundheitlichen Situation eine berufliche Veränderung in Erwägung (bzw. haben Sie diese bereits umgesetzt)?	☐	☐	☐	☐	☐
**53.**	Trifft es zu, dass Sie – im Vergleich mit anderen Menschen – größere Anstrengungen unternehmen müssen, um Ihre Ziele zu erreichen?	☐	☐	☐	☐	☐

VIELEN DANK FÜR IHRE WERTVOLLE UNTERSTÜTZUNG!
