# Supplementary material for: Development of a Social Network for People Without a Diagnosis (RarePairs): Evaluation Study
Source: J Med Internet Res. 2020 Sep 29;22(9):e21849. doi: 10.2196/21849 (PMC7556379; doi:10.2196/21849)
Supplement: Multimedia Appendix 4 [file jmir_v22i9e21849_app4.pdf]

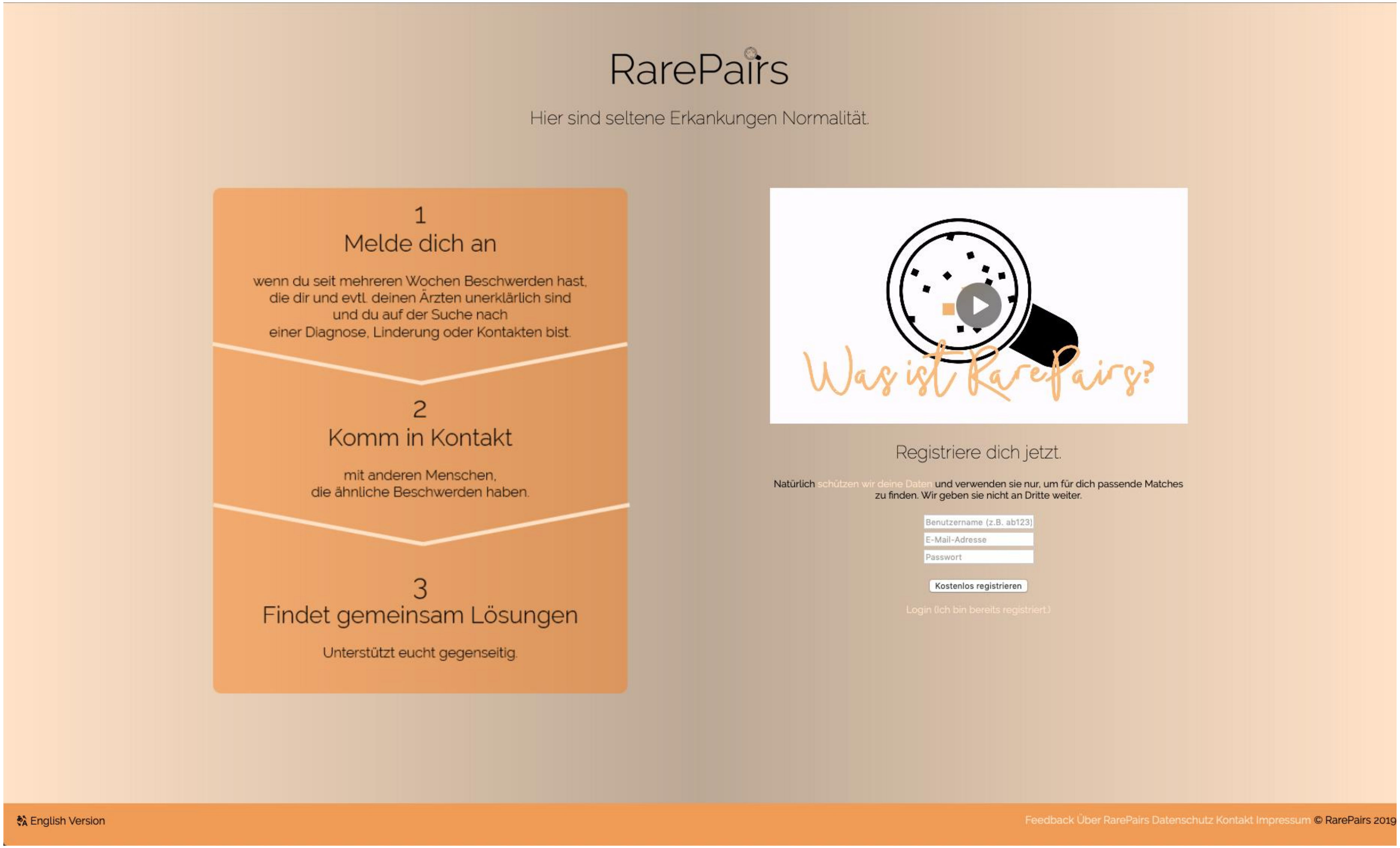

Landing Page of RarePairs where users can get information, register or log in. User find information by text and in form of a short video addressing aims and scope of RarePairs. Currently, the landing page is in German language.

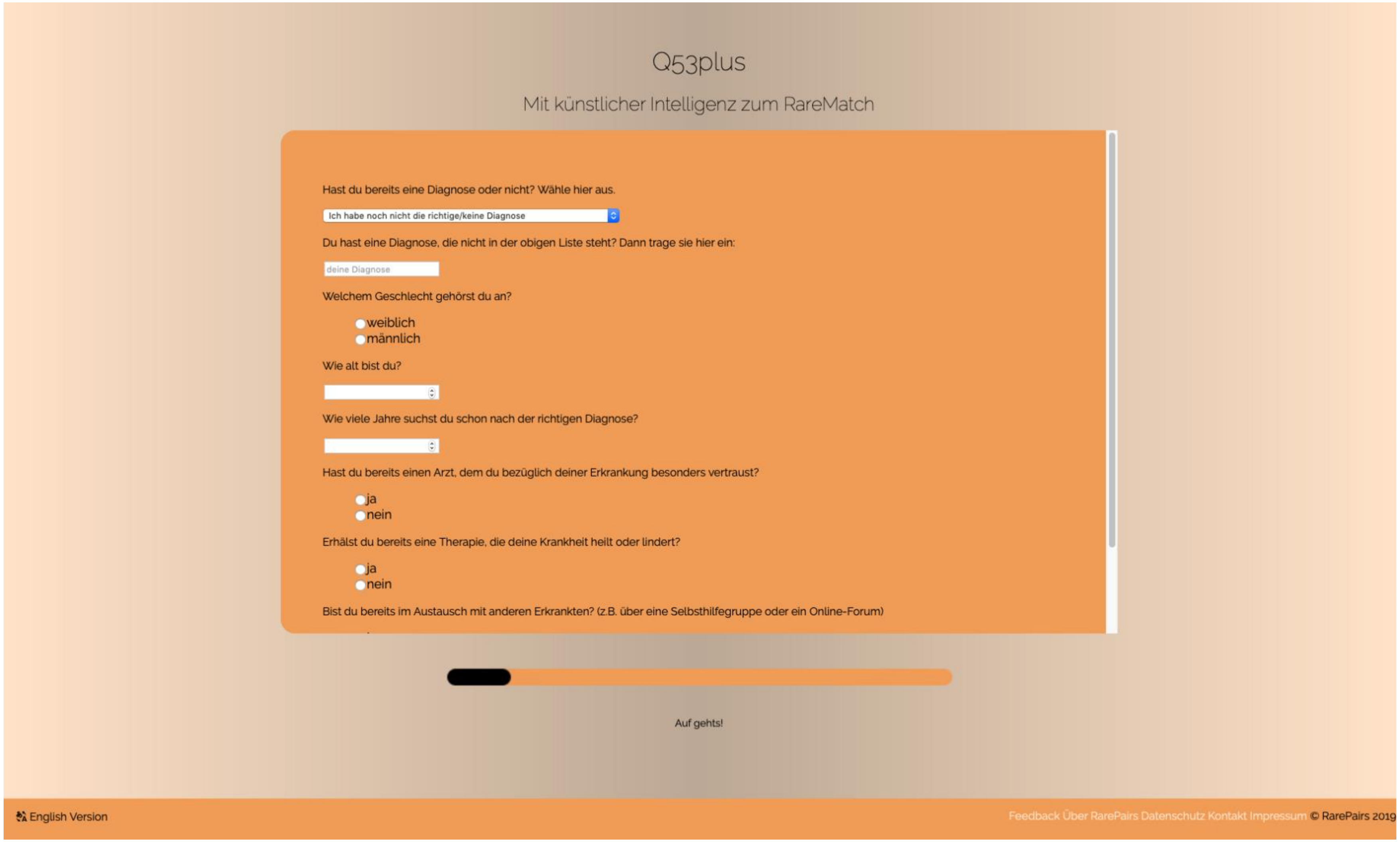

First page of the questionnaire adressing basic issues (. Is there already a diagnosis or not, is the user active in a patient group?, and age of the user). On the bottom of the page a user can see a progress bar.

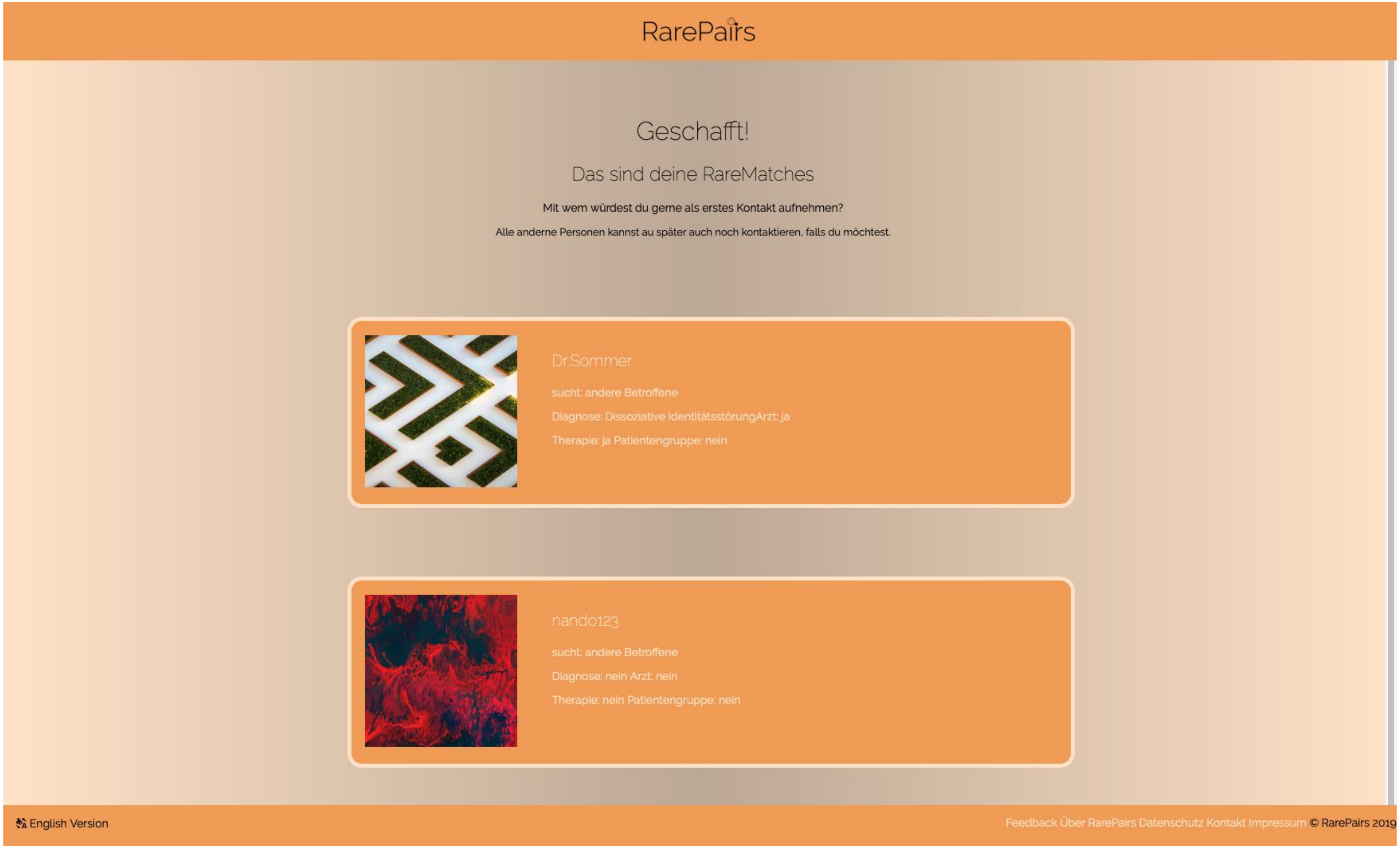

After completing the questionnaire the user can visualize his /her matches and has the option to contact them.

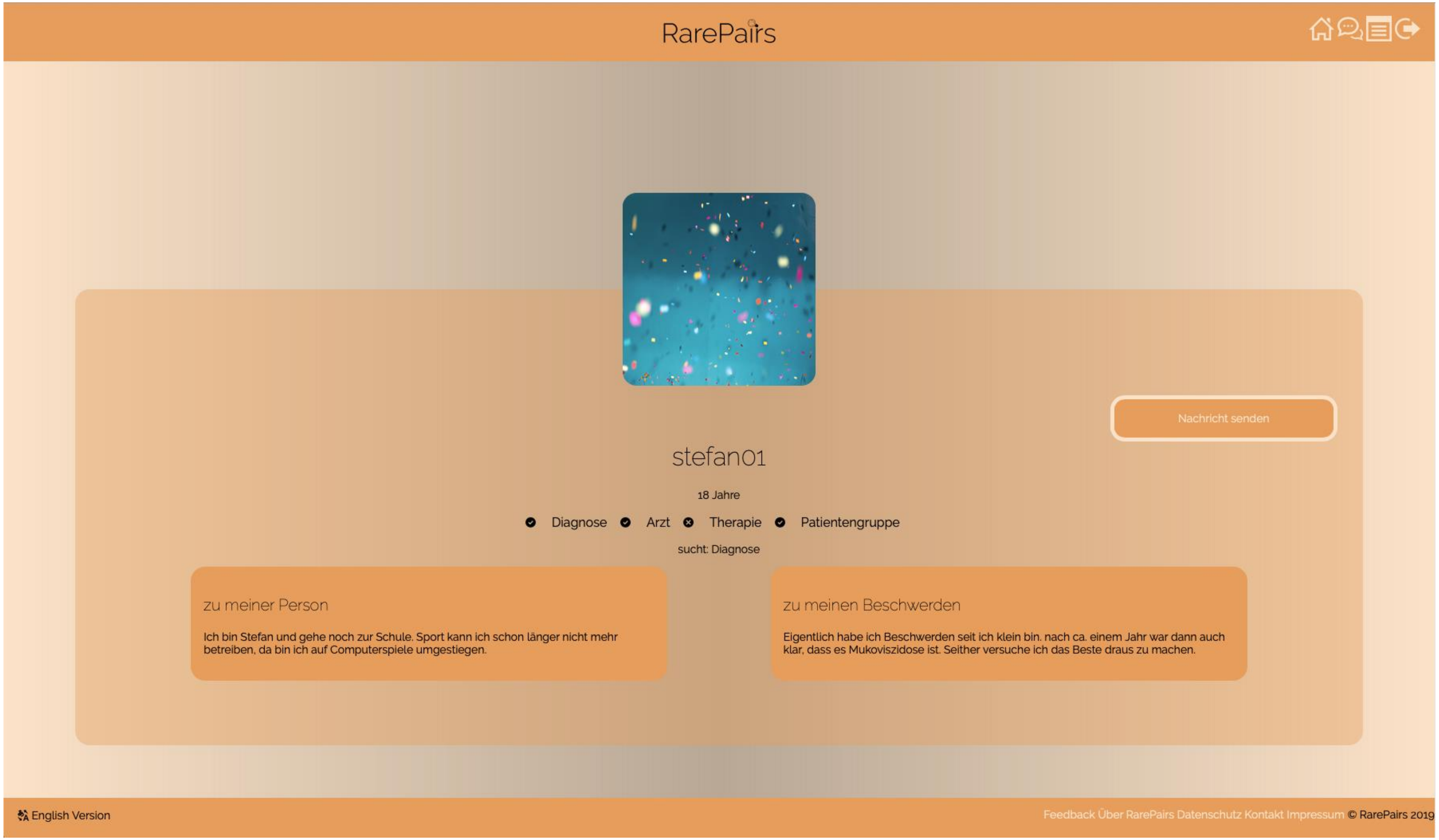

This screenshot shows the profile of another user in RarePairs.

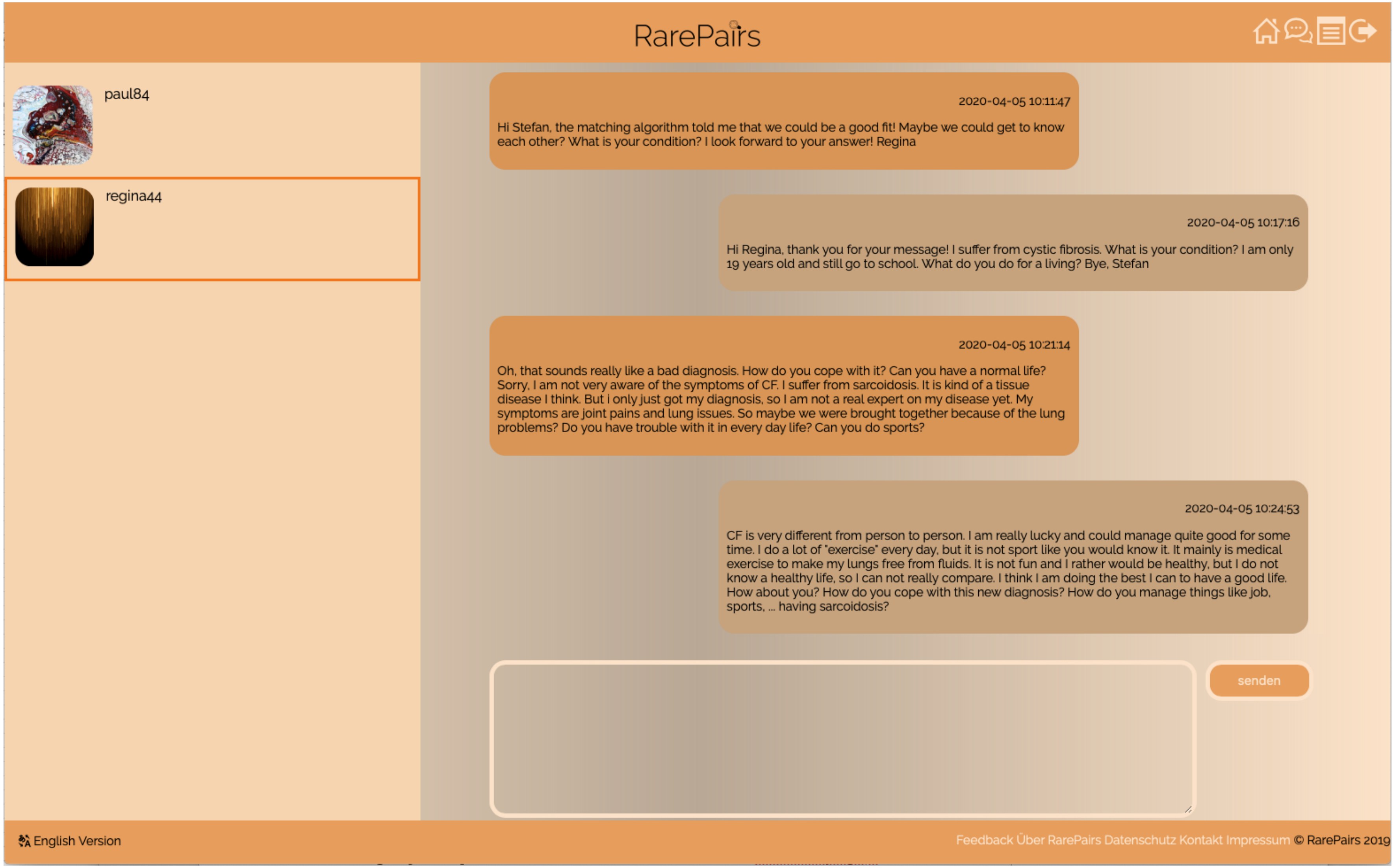

PairChat for communicating with one single person. On the left the users can find their current chat partners and on the right they can see all messages they exchanged with the selected chat partner.

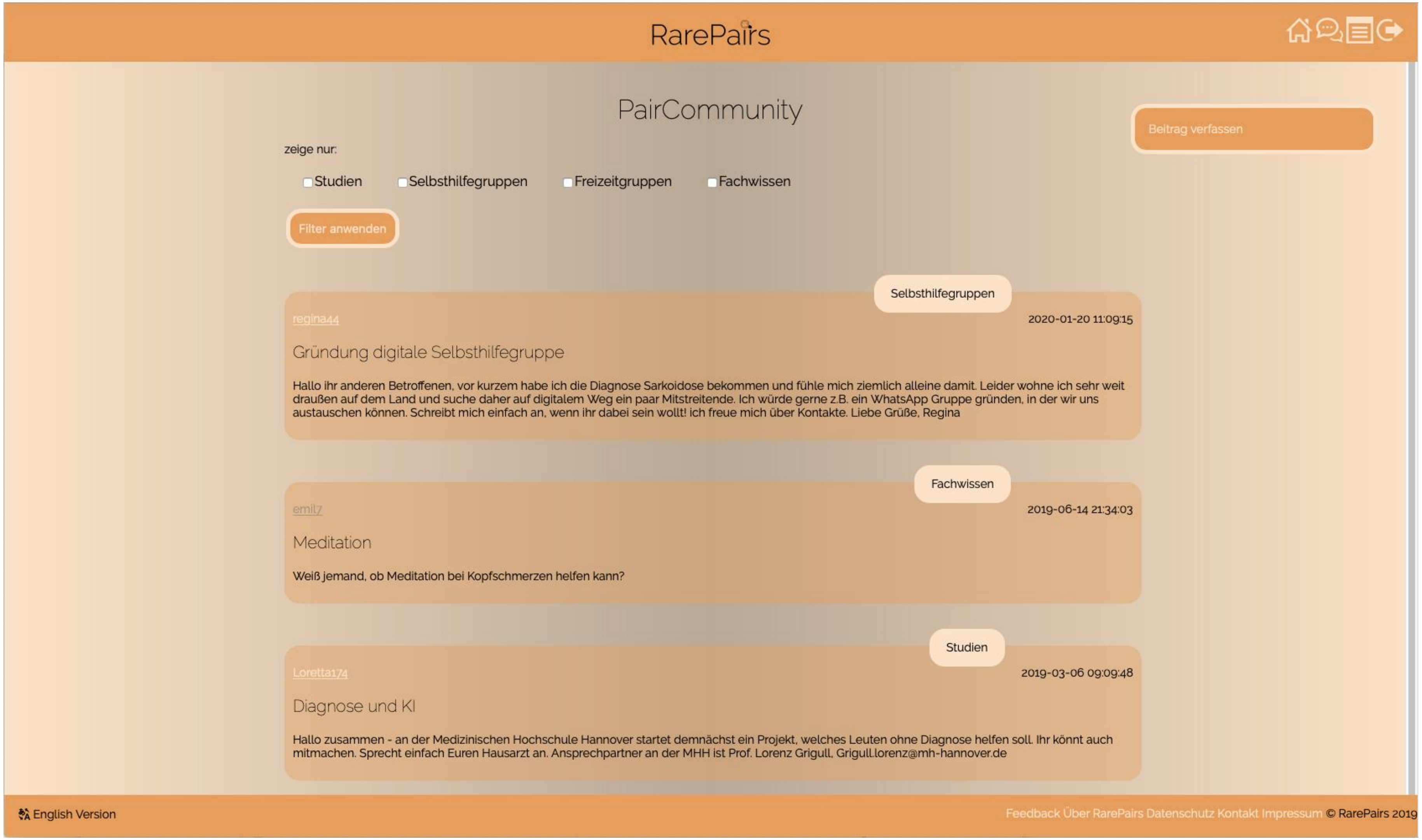

PairCommunity is the name of the forum where users can communicate with all other users at once. On the top, a filtering function is indicated. Here, users can choose to see only selected postings, e.g. current scientific studies or expert knowledge. Users can click on the button on the right to write their own postings and in the center of the page you can see all postings in chronological order.
