# Supplementary material for: Development of a Social Network for People Without a Diagnosis (RarePairs): Evaluation Study
Source: J Med Internet Res. 2020 Sep 29;22(9):e21849. doi: 10.2196/21849 (PMC7556379; doi:10.2196/21849)
Supplement: Multimedia Appendix 5 [file jmir_v22i9e21849_app5.pdf]

Manhattan $d{=}\sum_{i=1}^{d}|P_i-Q_i|$

Euclidean $d{=}\sqrt{\sum_{i=1}^{d}|P_i-Q_i|^2}$

Minkowski $d{=}\sqrt[p]{\sum_{i=1}^{d}|P_i-Q_i|^p}$

Sorensen $d{=}\frac{\sum_{i=1}^{d}|P_i-Q_i|}{\sum_{i=1}^{d}(P_i+Q_i)}$

Gower $d{=}\frac{1}{d}\cdot\sum_{i=1}^{d}|P_i-Q_i|$

Canberra $d{=}\sum_{i=1}^{d}\frac{|P_i-Q_i|}{P_i+Q_i}$

Lorentzian $d{=}\sum_{i=1}^{d}\ln\left(1+|P_i-Q_i|\right)$

Wave Hedges $d{=}\sum_{i=1}^{d}\frac{|P_i-Q_i|}{\max\left(P_i,Q_i\right)}$

Czekanowski $d{=}\frac{2\cdot\sum_{i=1}^{d}\min\left(P_i,Q_i\right)}{\sum_{i=1}^{d}(P_i+Q_i)}$

Tanimoto $d{=}\frac{\sum_{i=1}^{d}(\max\left(P_i,Q_i\right)-\min\left(P_i,Q_i\right))}{\sum_{i=1}^{d}\max\left(P_i,Q_i\right)}$

Jaccard $d{=}\frac{\sum_{i=1}^{d}(P_i-Q_i)^2}{\sum_{i=1}^{d}P_i^2+\sum_{i=1}^{d}Q_i^2-\sum_{i=1}^{d}P_i\cdot Q_i}$

Dice $d{=}\frac{\sum_{i=1}^{d}(P_i-Q_i)^2}{\sum_{i=1}^{d}P_i^2+\sum_{i=1}^{d}Q_i^2}$

Cosine $d{=}\frac{\sum_{i=1}^{d}P_i\cdot Q_i}{\sqrt{\sum_{i=1}^{d}P_i^2}\cdot\sqrt{\sum_{i=1}^{d}Q_i^2}}$

Bhattacharyya $d{=}-\ln\sum_{i=1}^{d}\sqrt{P_i\cdot Q_i}$

Hellinger $d{=}2\cdot\sqrt{1-\sum_{i=1}^{d}\sqrt{P_i\cdot Q_i}}$

Squared-Chord $=\sum_{i=1}^{d}(\sqrt{P_i}-\sqrt{Q_i})^2$

Neyman $d{=}\sum_{i=1}^{d}\frac{(P_i-Q_i)^2}{P_i}$

Probabilistic Symmetric $d{=}2\cdot\sum_{i=1}^{d}\frac{(P_i-Q_i)^2}{P_i+Q_i}$

Clark $d{=}\sqrt{\sum_{i=1}^{d}(\frac{|P_i-Q_i|}{P_i+Q_i}})^2$

Additive Symmetric $d{=}\sum_{i=1}^{b}\frac{(P_i-Q)^2\cdot(P_i+Q_i)}{P_i\cdot Q_i}$

Jeffreys $d{=}\sum_{i=1}^{d}\left(P_i-Q_i\right)\cdot\ln\frac{P_i}{Q_i}$

Jensen difference $d{=}\sum_{i=1}^{b}((\frac{P_i\cdot\ln P_i+Q_i\cdot\ln Q_i}{2})-(\frac{P_i+Q_i}{2})\cdot\ln(\frac{P_i+Q_i}{2}))$

Kumar-Johnson $d = \sum_{i=1}^{d} \frac{(P_i^2 - Q_i^2)^2}{2 \cdot (P_i \cdot Q_i)^{\frac{3}{2}}}$

Avg $d = \frac{\sum_{i=1}^{d} |P_i - Q_i| + \max |P_i - Q_i|}{2}$
