# Supplementary material for: Development of a Social Network for People Without a Diagnosis (RarePairs): Evaluation Study
Source: J Med Internet Res. 2020 Sep 29;22(9):e21849. doi: 10.2196/21849 (PMC7556379; doi:10.2196/21849)
Supplement: Multimedia Appendix 6 [file jmir_v22i9e21849_app6.pdf]

	Accordance in Percent					
**Distance calculating method**	**Gender**	**Age**	**Latency**	**Disease group**	**Diagnostic system**	**Exact diagnosis**
(Pseudo-)random sampling (=negative benchmark)	59.640	15.704	11.850	8.304	31.716	3.813
**L_p Minkowski family**						
Manhattan	63.864*	21.871*	14.317	16.218*	40.298*	9.383*
Euclidean	62.487*	21.161	15.447*	15.982*	36.105*	**9.712** (=positive benchmark)
Minkowski	63.823*	21.881*	14.358	16.218*	40.298*	9.383*
**L_1 family**						
Sørensen	64.810*	22.107*	13.196	15.324*	37.780*	8.767
Gower	63.823*	21.881*	14.358	16.218*	40.298*	9.383*
Canberra	64.162*	21.531*	13.957	15.817*	37.646*	9.353* (does not differ from pos. benchmark; p = .56)
Lorentzian	63.546*	21.727*	13.936	15.889*	39.404*	9.322*
**Intersection family**						
Wave Hedges	64.049*	21.490*	13.977	15.899*	38.253*	9.322
Czekanowski	64.810*	22.107*	13.196	15.324*	37.780*	8.767

Tanimoto	**64.810** (=positive benchmark)	**22.107** (=positive benchmark)	13.196 (differs from neg. benchmark; p = .00)	15.324*	37.780*	8.767
Jaccard	63.782*	21.305	13.823	14.892	35.396*	8.715
Dice	63.782*	21.305 (differs from pos. benchmark; p = .03)	13.823	14.892 (differs from pos. benchmark; p = .05)	35.396*	8.715 (differs from neg. benchmark; p = .00)
**Inner Product family**						
Cosine	53.587# (< neg. benchmark)	11.973# (< neg. benchmark)	7.307# (< neg. benchmark)	4.697# (< neg. benchmark)	16.978# (< neg. benchmark)	1.932# (< neg. benchmark)
**Fidelity family or Squared-chord family**						
Bhattacharyya	64.769*	19.003 (differs from neg. benchmark; p = .00)	7.914# (< neg. benchmark)	10.062# (does not differ from neg. benchmark; p = .70)	32.312 (differs from neg. benchmark; p = .00; differs from pos. benchmark; p = .00)	2.806# (< neg. benchmark)
Hellinger	62.929*	19.291	5.807# (< neg. benchmark)	7.441# (< neg. benchmark)	**52.467** (=positive benchmark)	0.308# (< neg. benchmark)
Squared-Chord	61.984*	21.603*	15.334*	15.632*	35.200*	9.609*
**Squared L_2 family dX_2 family**						

Neyman	59.496# (< neg. benchmark)	20.411	15.211 (differs from pos. benchmark; p = .02)	14.892 (differs from neg. benchmark; p = .00)	34.234*	9.024
Probabilistic Symmetric	62.220*	21.634*	15.128	15.817*	35.478*	9.681*
Clark	63.073*	21.439* (does not differ from pos. benchmark; p = .42)	14.830	15.334*	35.488*	9.322
Additive Symmetric	61.254* (does not differ from pos. benchmark; p = .08)	21.059	15.683*	15.447*	34.090*	9.517*
**Shannon's entropy family**						
Jeffreys	61.973*	21.490*	15.313*	15.622*	35.108*	9.599*
Jensen difference	62.076*	21.531*	15.293* (does not differ from pos. benchmark; p = .55)	15.683*	35.303*	9.630*
**Combined methods**						
Kumar-Johnson	60.791 (differs from pos. benchmark; p = .02)	21.213	**15.725** (=positive benchmark)	15.036* (does not differ from pos. benchmark; p = .08)	33.371* (does not differ from pos. benchmark; p = .14)	9.332* (differs from pos. benchmark; p = .02)

Avg	63.720	21.778#	14.430	**16.362** (=positive benchmark)	40.298*	9.414*

legend

gender = gender of the person;

age = age of the person;
latency = time with symptoms but no diagnosis;
disease group = category of the diagnosis referring to the affected organ or pathophysiology (e.g. neuromuscular disease, metabolic disease);
diagnostic system = greater category the diagnosis can be assigned to (e.g. RD, CD) not especially considering the affected organ; exact diagnosis = exact name of the one diagnosis.

* Fields do not differ significantly from the positive benchmark in this category; see p value in those fields or if no p values are mentioned the matching- values lie in between the benchmark and the furthest value which is only just not differing significantly from this benchmark
# Fields do not differ significantly from the (pseudo-) random matching (=negative benchmark); see p value in those fields or if no p values are mentioned the matching- values lie in between the benchmark and the furthest value which is only just not differing significantly from this benchmark.
